# Risk accuracy of type 2 diabetes in middle aged adults: Associations with sociodemographic, clinical, psychological and behavioural factors

**DOI:** 10.1016/j.pec.2017.07.023

**Published:** 2018-01

**Authors:** Barbora Silarova, Fiona E. Douglas, Juliet A. Usher-Smith, Job G. Godino, Simon J. Griffin

**Affiliations:** a MRC Epidemiology Unit, University of Cambridge, Institute of Metabolic Science, Cambridge, CB2 0QQ, UK; b School of Clinical Medicine, University of Cambridge, Box 111 Cambridge Biomedical Campus, Cambridge, CB2 0SP, UK; c The Primary Care Unit, Department of Public Health and Primary Care, University of Cambridge, Strangeways Research Laboratory, 2 Wort’s Causeway, Cambridge, CB1 8RN, UK; d Center for Wireless and Population Health Systems, University of California, San Diego, 9500 Gilman Drive, La Jolla, CA 92093-0811, USA

**Keywords:** Communication, Diabetes mellitus, type 2, Optimism, Risk assessment

## Abstract

**Objective:**

To identify the proportion of individuals with an accurate perception of their risk of type 2 diabetes (T2D) prior to, immediately after and eight weeks after receiving a personalised risk estimate. Additionally, we aimed to explore what factors are associated with underestimation and overestimation immediately post-intervention.

**Methods:**

Cohort study based on the data collected in the Diabetes Risk Communication Trial. We included 379 participants (mean age 48.9 (SD 7.4) years; 55.1% women) who received a genotypic or phenotypic risk estimate for T2D.

**Results:**

While only 1.3% of participants perceived their risk accurately at baseline, this increased to 24.7% immediately after receiving a risk estimate and then dropped to 7.3% at eight weeks. Those who overestimated their risk at baseline continued to overestimate it, whereas those who underestimated their risk at baseline improved their risk accuracy. We did not identify any other characteristics associated with underestimation or overestimation immediately after receiving a risk estimate.

**Conclusion:**

Understanding a received risk estimate is challenging for most participants with many continuing to have inaccurate risk perception after receiving the estimate.

**Practice implications:**

Individuals who overestimate or underestimate their T2D risk before receiving risk information might require different approaches for altering their risk perception.

## Highlights

• Most people overestimate their T2D risk before they received a risk estimate.
• Those who overestimated their T2D risk at baseline continued to overestimate it after receiving a risk estimate.
• Those who underestimated their T2D risk at baseline improved their risk accuracy after receiving a risk estimate.

## 1 Introduction

An estimated 382 million people worldwide had type 2 diabetes (T2D) in 2013 [1], and this number is expected to increase by more than 55% by 2035 if effective preventive strategies are not implemented. A key part of many primary preventive strategies, including the NHS Diabetes Prevention Programme [2], is identification of individuals who are at high risk of developing T2D who can be referred to lifestyle intervention services to reduce their risk [3], [4].

Some theories of behaviour change [5], [6], [7], [8] hypothesise that behavioural interventions may only be successful if individuals perceive themselves to be at risk of developing a disease. This is reflected in the recommendation from the UK National Institute for Health and Care Excellence (NICE) that if health professionals assess an individual’s T2D risk, they should communicate it to their patients [9]. Similarly, Diabetes UK encourages the public to find out their risk through the Know Your Risk campaign [10]. While there is limited research directly on this topic, systematic reviews in the context of cardiovascular disease and cancer have shown that providing people with risk information does not alter risk perception among all participants equally [11], [12]. For example, even immediately after being provided with cardiovascular risk information, one in four participants still have an inaccurate perceived risk [13] and one in ten change their perceived risk in the opposite direction to the feedback they receive [14]. Provision of cancer risk information also modifies patients’ risk perceptions only in specific subgroups (e.g. when focusing on specific types of cancer or specific formats of risk) [12]. Additionally, in a recent RCT provision of risk information improved risk accuracy at 6 month follow-up among those who originally underestimated their risk for T2D, yet the majority of those who underestimated their risk did not correct their risk perception [15].

A limited number of cross-sectional studies have explored individuals’ accuracy of perceived risk of T2D and longitudinal studies seem to be even rarer [15]. Little is also known about the characteristics of those, who after the provision of risk information, perceive their T2D risk accurately and those who, even after they receive information on their risk, still underestimate or overestimate their risk. Identifying such characteristics will improve our understanding of the potential impact of risk communication and inform development of more effective interventions and health promotion campaigns targeted at changing perceived T2D risk.

We aimed to quantify the prevalence of accurate risk perception, underestimation and overestimation of individuals’ T2D risk at three time points: baseline (before the provision of risk information), immediately and eight weeks after provision of risk information. Additionally, we aimed to explore which factors (e.g. personal characteristics and behavioural risk factors) are associated with underestimation and overestimation immediately after provision of risk information.

## 2 Methods

### 2.1 Participants and setting

The present study is a cohort study based on the data collected at baseline, immediately post-intervention and eight weeks post-intervention in the Diabetes Risk Communication Trial (DRCT). The study design is described in more detail elsewhere [16]. In summary, the DRCT was an open randomised controlled trial investigating the effect of provision of diabetes risk information on physical activity levels [16]. Participants were allocated in a ratio of 1:1:1 to three study arms: participants in the control group received standard lifestyle advice about T2D and risk reducing behaviours and participants in the active intervention groups received either a genetic risk estimate for T2D or a phenotypic risk estimate for T2D in addition to standard lifestyle advice [16], [17]. The risk-based intervention was grounded in Protection Motivation Theory (PMT) [18], and the Common Sense Model (CSM) of self-regulation was used to guide understanding of how people construct a perceived risk [19]. The standard lifestyle advice was part of the booklet that also included information on T2D, risk factors, symptoms, diagnosis, and consequences of the disease reflecting determinants of health behaviours as postulated by PMT and CSM.

Participants in the DRCT study were recruited from the ongoing population-based Fenland study [20]. They were born between 1950 and 1975, were registered with participating general practices in Cambridgeshire, UK, had enough data available to enable calculation of their genetic and phenotypic risk of T2D, and wore a physical activity monitor for at least three full days which provided at least 36 h of complete physical activity data [16]. Participants were excluded if they had diagnoses of diabetes, a terminal illness with a prognosis of less than one year or a psychotic illness, were pregnant or lactating, or were unable to walk unaided at the time of recruitment [16]. The study obtained full ethical approval from the Cambridgeshire 1 Research Ethics Committee (10/H0304/78) on 21st October 2010. All participants provided written informed consent.

### 2.2 Measures

#### 2.2.1 Risk accuracy

Participants’ risk accuracy was measured at baseline, immediately post-intervention and eight weeks post-intervention. The baseline assessment occurred before randomisation, and after participants were allocated to the study arm, they were mailed standard lifestyle advice with a risk estimate (either phenotypic or genetic) and were instructed to immediately complete a post-intervention questionnaire. Participants were instructed to read through the intervention materials, complete the questionnaire and return it to the study team. Participants received a reminder letter if the study team did not receive the post-intervention questionnaire within two weeks [16].

**Fig. 1:**
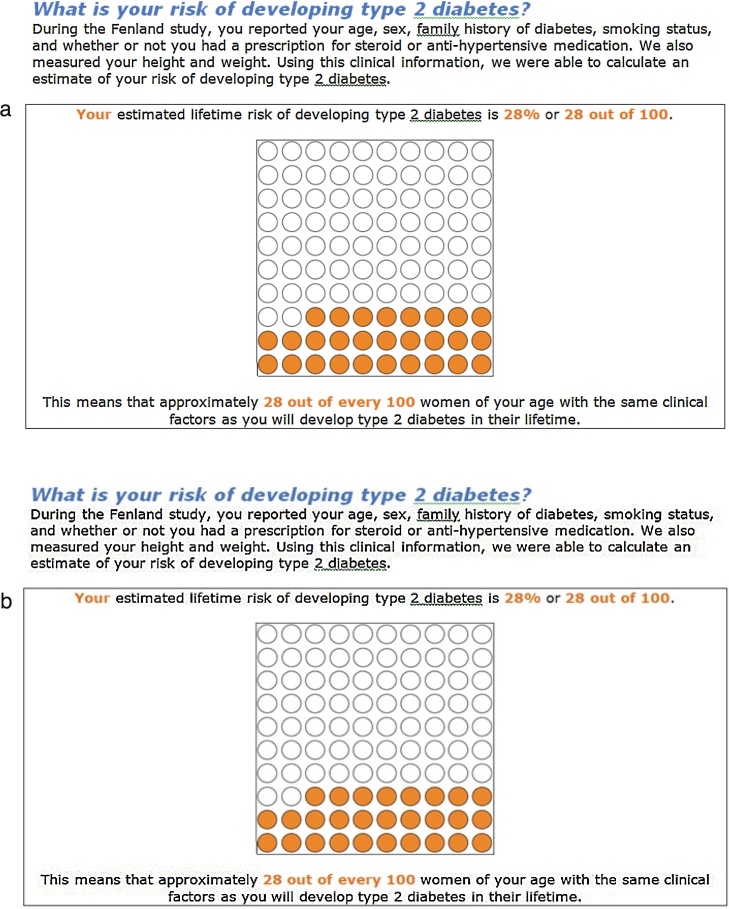
(a) An example of how modelled absolute lifetime type 2 diabetes phentoypic risk was communicated to participants (b) An example of how modelled absolute lifetime type 2 diabetes genetic risk was communicated to participants.

To determine risk accuracy, we matched participants’ self-reported absolute perceived lifetime risk of T2D to their modelled absolute lifetime T2D risk. Perceived risk was assessed with the question, ‘On a scale from 0 to 100, where 0 = certain not to happen, and 100 = certain to happen, how likely are you to get type 2 diabetes in your lifetime?’ This item had been adapted according to recommendations by Diefenbach et al. [21] and generally reflected the epidemiological communication of risk at the time of designing DRCT trial [22], [23]. Modelled absolute lifetime T2D risk was assessed using the previously validated Cambridge Diabetes Risk score [24] (see below) and communicated to participants as a percentage (whole number, i.e. no decimal places) and pictorial risk of T2D (Fig. 1a,b). In the analyses, we used risk accuracy as both a continuous variable and categorical variable. Risk accuracy as a continuous variable was defined as the difference between perceived risk and modelled absolute lifetime T2D risk. For the categorical risk accuracy variable, we grouped participants into three categories: ‘accurate’ (those who accurately reported their risk); ‘underestimate’ (those reporting a perceived lifetime risk lower than they were told) and ‘overestimate’ (those reporting a higher perceived lifetime risk than they were told). ‘Accurate’ risk perception was defined as a perceived risk that equalled the communicated risk (difference of 0).

#### 2.2.2 Socio-demographic, clinical and psychological factors

Socio-demographic factors (age, sex, race/ethnicity, employment status, level of education, annual household income and marital status) were measured by self-reported questionnaire during the Fenland study.

We grouped race/ethnicity as ‘white’ and ‘other ethnic background’. We categorised employment status as ‘not working’, ‘part-time’ and ‘full-time’. Level of education was recorded as age at which the participant finished education and was treated as a continuous variable. We grouped annual household income into ‘Less than £20,000′; ‘Between £20,000 and £40,000′; and ‘More than £40,000′ (i.e. the poverty line in the UK for 2014 to 2015 for a couple with 2 children, was £20,436 per year, [25]). Marital status was categorised into ‘Married or living as married’ and ‘other’.

The Diabetes Risk score, genetic risk estimate, a family history of T2D, and smoking status were calculated or assessed using data collected during the Fenland study. The Diabetes Risk score was estimated at baseline before participants were randomised into study arms. It is an estimate of the individual’s lifetime risk of developing T2D calculated using the Cambridge Diabetes Risk Score which includes age, sex, body mass index (BMI), smoking status, family history of diabetes and prescription of steroid or anti-hypertensive medication [24]. The Diabetes Risk score ranges from 0 to 100. Genetic risk estimate was also estimated at baseline before participants were randomised into study arms. It was calculated following similar steps to those published by several direct to consumer genetic testing companies and ranged from 0 to 100 [16]. A positive family history of T2D was defined as having at least one parent or sibling with T2D. We grouped smoking status as ‘current smoker’ and ‘never smoked/ex-smoker’. Behavioural intentions to be physically active and maintain a healthy diet were assessed at baseline, each using 2 items. Participants provided their responses on a five-point Likert scale. These measures were used similarly in T2D research previously [26]. The total score for behavioural intentions for diet/physical activity was calculated as a sum of two items divided by two. The total scores range from 1 to 5. Response efficacy for diet and physical activity was measured using a self-reported questionnaire at baseline, using two items with five-point Likert scales. They assessed the belief that diet or physical activity can reduce the risk of developing T2D [27]. The total score for response efficacy for diet/physical activity was calculated as a sum of two items divided by two. The total scores range from 1 to 5. Self-efficacy for physical activity and diet was also measured by self-reported questionnaire at baseline, using two items with five-point Likert scales. It represents an individual’s belief that they are able to modify their behaviour [28]. The total score for self-efficacy for diet/physical activity was calculated as a sum of two items divided by two. The total scores range from 1 to 5.

#### 2.2.3 Health behaviours

Both physical activity and fruit and vegetable consumption were measured at baseline in the Fenland study. Physical activity was measured objectively via a combined heart rate monitor and accelerometer (Actiheart^®^) and defined as physical activity energy expenditure (PAEE, measured in kJ/kg/day) [29]. Participants were asked to wear an Actiheart^®^ monitor for six days and nights continuously. As monitors are also waterproof, they could be worn while swimming or showering. Fruit and vegetable intake (g/day) was assessed through self-report using the Food Frequency Questionnaire which contains a list of 130 foods [30], [31] including 12 fruit items and 26 vegetable items.

### 2.3 Statistical analysis

We conducted all statistical analyses using IBM SPSS Statistics for Windows, version 21.0 (IBM company, Chicago, Illinois, USA). Those with missing data were excluded from the analyses. As there were no differences in risk perception between those receiving genetic risk information and phenotypic risk information immediately post-intervention and eight weeks post-intervention [17], we combined participants into one group. Given the number of comparisons we considered the results as convincing if the 2 sided *p*-value was <0.001. As a first step, we summarised normally distributed continuous variables as means and standard deviations (SD), skewed continuous variables as medians and interquartile range (IQR), and categorical variables as the number and percentage of participants within each category. Next, we used box plots to display the distribution of risk accuracy at different time points (baseline, immediately post-intervention and eight weeks post-intervention). We also tested differences between risk accuracy measured at different time points using related-samples Wilcoxon signed rank tests. Finally, we performed univariate multinomial logistic regression analyses to explore whether socio-demographic, clinical and psychological factors, as well as health behaviours were associated with different categories of risk perception (accurate/underestimate/overestimate) measured immediately post-intervention. Accurate risk perception was set as a reference category. We also performed sensitivity analysis, in which ‘accurate’ was defined as a perceived risk that is within the ±5% of communicated risk.

## 3 Results

### 3.1 Characteristics of the sample

The baseline characteristics of the sample are presented in Table 1. In the DRCT, 569 participants were randomised into the three study arms. For the purpose of this study, we included only participants (*n* = 379) who were allocated to the two active intervention arms and therefore received a genetic or phenotypic risk estimate for T2D. The mean age of the present sample was 48.9 (SD 7.4) years with slightly more women than men (55.1% vs 44.9%). The majority of the sample reported white ethnicity (95.2%) and had a fulltime job (67.6%). The median lifetime Diabetes risk was 22.9% (IQR 13.9) and 39 (10.3%) participants were current smokers.

**Table 1:** Characteristics of the Sample.

Variable	Total	Median(IQR)/n(%)
Age (years): mean (SD)	379	48.9 (SD 7.4)
Sex	379	
Female		209 (55.1%)
Race/Ethnicity	375	
White		357 (95.2%)
Other ethnic background		18 (4.8%)
Employment status	370	
Not working		42 (11.4%)
Part-time		78 (21.1%)
Fulltime		250 (67.6%)
Age when finished education (years)	372	18 (IQR 6.0)
Annual household income	370	
Less than £20,000		53 (14.3%)
Between £20,000 and £40,000		130 (35.1%)
More than £40,000		187 (50.5%)
Marital status		
Married or living as married		284 (81.8%)
Other		63 (18.2%)
Diabetes Risk Score (0–100)	379	22.9 (IQR 13.9)
Family history of diabetes	379	
Yes		88 (23.2%)
Smoking status	369	
Never smoked/ex-smoker		331 (89.7%)
Current smoker		39 (10.3%)
Fruit and vegetable intake (g/day)	378	394 (IQR 264.1)
Physical activity (KJ/kg/day): mean (SD)	378	52.8 (SD 21.1)
Behavioural intention (1–5)		
Diet	378	3.5 (IQR 1.0)
Physical activity	378	3.5 (IQR 1.0)
Response efficacy (1–5)		
Diet (less or equal 4)	378	273 (72.2%)
Physical activity (less or equal 4)	377	291 (77.2%)
Self-efficacy (1–5)		
Diet (less or equal 4)	378	273 (72.2%)
Physical activity (less or equal 4)	378	275 (72.8%)
Study arm	379	
Phenotypic risk		190 (50.1%)
Genetic risk		189 (49.9%)

IQR − interquartile range; SD − standard deviation.

NOTE: only non-missing data are presented.

### 3.2 Risk accuracy

**Fig. 2:**
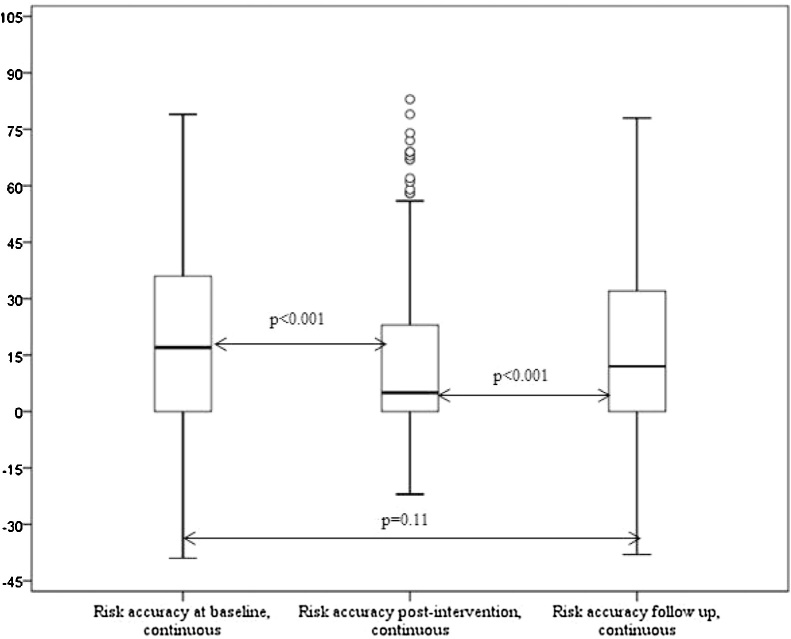
Risk accuracy (continuous) at baseline, immediately post-intervention and eight weeks follow-up: total sample. Legend: The boxplots present the minimum, the lower quartile, the median, the upper quartile and the maximum. There were significant differences between risk accuracy at baseline and risk accuracy immediately post-intervention (p < 0.001; Related −Samples Wilcoxon Signed Rank Test) and between risk accuracy immediately post-intervention and risk accuracy at eight weeks follow-up (p < 0.001; Related −Samples Wilcoxon Signed Rank Test). There were no significant differences between risk accuracy at baseline and risk accuracy at eight weeks follow-up (p = 0.113; Related −Samples Wilcoxon Signed Rank Test).

Table 2 and Fig. 2 show the risk accuracy at baseline, immediately post intervention and eight weeks post intervention for the total sample. 20 (5.3%) of participants had missing information on risk accuracy either at baseline, immediately post-intervention or at eight weeks post-intervention. There were no significant differences in any of the characteristics presented in Table 1 between those with a missing information on risk accuracy either at baseline, immediately post-intervention or at eight weeks post-intervention and those without a missing information on risk accuracy. Most people did not perceive their risk accurately at baseline (74.5% overestimated and 24.1% underestimated their risk). While only 1.3% of participants perceived their risk accurately at baseline, this increased to 24.7% immediately after receiving a risk estimate and then dropped to 7.3% at eight weeks. Related-samples Wilcoxon signed rank tests showed significant differences between risk accuracy at baseline and risk accuracy at post-intervention (p < 0.001) and between risk accuracy at post-intervention and eight weeks follow-up (p < 0.001). There was no significant difference between risk accuracy at baseline and risk accuracy at eight weeks follow-up (p = 0.11). The sensitivity analyses (in which ‘accurate’ was defined as a perceived risk that is within the ±5% of communicated risk) showed the same pattern of associations.

**Table 2:** Risk accuracy at baseline, immediately post-intervention and eight weeks post-intervention.

	Total	Median (IQR)	n (%)^a^	n (%)^b^
Baseline − cont.	373	17 (IQR 36.0)		
Baseline − cat.	373			
Accurate			5 (1.3%)	48 (12.9%)
Underestimate			90 (24.1%)	69 (18.5%)
Overestimate			278 (74.5%)	256 (68.6%)

Immediately post intervention − cont.	365	5 (IQR 24.0)		
Immediately post intervention − cat.	365			
Accurate			90 (24.7%)	150 (41.1%)
Underestimate			61 (16.7%)	35 (9.6%)
Overestimate			214 (58.6%)	180 (49.3%)
				
Eight weeks post intervention– cont.	368	12 (IQR 32.0)		
Eight weeks post intervention – cat.	368			
Accurate			27 (7.3%)	100 (27.2%)
Underestimate			78 (21.2%)	43 (11.7%)
Overestimate			263 (71.5%)	225 (61.1%)

Risk accuracy calculated as the difference between self-reported perceived lifetime risk of type 2 diabetes and diabetes risk score given to the participant.

NOTE: only non-missing data are presented.

a Accurate defined as a perceived risk that equalled the communicated risk.

b Accurate defined as a perceived risk that is within ±5% of the communicated risk.

**Fig. 3:**
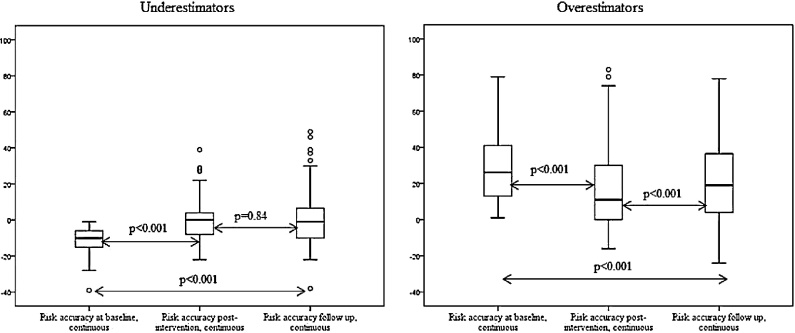
Risk accuracy (continuous) at baseline, immediately post-intervention and eight weeks follow-up: underestimators and overestimators. Legend: The boxplots present the minimum, the lower quartile, the median, the upper quartile and the maximum. Those who underestimated their risk at baseline, perceived their risk more accurately immediately post-intervention (p < 0.001; Related −Samples Wilcoxon Signed Rank Test) and continued to perceive their risk accurately at eight weeks follow-up (p = 0.84; Related −Samples Wilcoxon Signed Rank Test). Those who overestimated their T2D risk at baseline, improved the accuracy of their risk perception immediately post-intervention but still overestimated their risk (p<0.001; Related −Samples Wilcoxon Signed Rank Test). At eight weeks follow-up they more inaccurate than immediately post-intervention but less than at baseline.

Further exploration of patterns in risk accuracy (Table 3 and Fig. 3), shows that those who underestimated their risk at baseline perceived their risk more accurately immediate post-intervention (p < 0.001; Related −Samples Wilcoxon Signed Rank Test) and continued to perceive their risk more accurately at eight weeks post-intervention (p = 0.84; Related −Samples Wilcoxon Signed Rank Test). By comparison, those who overestimated their T2D risk at baseline perceived their risk more accurately immediately post-intervention but still overestimated their risk (p < 0.001; Related −Samples Wilcoxon Signed Rank Test). At eight weeks follow-up they overestimated their risk more than immediately post-intervention but less than at baseline. The sensitivity analyses (in which ‘accurate’ was defined as a perceived risk that is within the ±5% of communicated risk) showed the same pattern of associations.

**Table 3:** Risk accuracy at baseline, immediately and eight weeks post-intervention: underestimators and overestimators.

	Underestimator at baseline			Overestimator at baseline		
	Total	Median (IQR)	n (%)^a^	Total	Median (IQR)	n (%)^a^
Risk accuracy at baseline − cont.	90	-10 (IQR 9.0)		278	26 (IQR 28.0)	
Risk accuracy immediately post intervention − cont.	87	0 (IQR 13.0)		270	11 (IQR 30.3)	
Risk accuracy immediately post intervention −cat.	87			270		
Accurate			21 (24.1%)			62 (23.0%)
Underestimate			37 (42.5%)			24 (8.9%)
Overestimate			29 (33.3%)			184 (68.1%)
Risk accuracy at eight weeks post-intervention −cat.	87	-1 (IQR 18.0)		271	19.0 (IQR 33.0)	
Risk accuracy at eight weeks post-intervention −cat.	87			271		
Accurate			9 (10.3%)			15 (5.5%)
Underestimate			48 (55.2%)			28 (10.3%)
Overestimate			30 (34.5%)			228 (84.1%)

Risk accuracy calculated as the difference between self-reported perceived lifetime risk of type 2 diabetes and diabetes risk score given to the participant.

NOTE: only non-missing data are presented.

a Accurate defined as a perceived risk that equalled the communicated risk.

### 3.3 Correlates of underestimate and overestimate risk perception

Socio-demographic, clinical, and psychological factors as well as health behaviours potentially associated with underestimation and overestimation of risk of T2D immediately post provision of risk information are shown in Table 4. We did not find any statistically significant associations (p < 0.001). There was an indication that those who finished their education at a higher age were less likely to underestimate and overestimate their risk ((OR (95%CI): 0.93 (0.87–0.998)) and 0.89 (0.84–0.94) respectively) (Table 4). Sensitivity analyses (in which ‘accurate’ was defined as a perceived risk that is within the ±5% of communicated risk) showed that age when finished education was significantly associated (p < 0.001) only with overestimation of risk (OR (95%CI): 0.91 (0.86–0.97)). There was also an indication that those participants who received a higher risk estimate were more likely to underestimate their risk (OR (95%CI): 1.05 (1.01–1.08)) and this reached statistical significance (p < 0.001) when we ran sensitivity analyses (OR (95%CI): 1.04 (1.01–1.06)). Lastly there was an indication that those who have higher BMI were more likely to overestimate their risk of T2D (OR (95%CI):1.13 (1.05–1.20)), this also reached statistical significance (p < 0.001) when we ran a sensitivity analysis (OR (95%CI):1.11 (1.05–1.17)).

**Table 4:** Correlates of Risk accuracy immediately post-provision of risk information.

Variable	Total	Underestimate vs Accurate	Overestimate vs Accurate
		OR (95%CI)	OR (95%CI)
Age (years)	365	0.99 (0.95–1.04)	1.01 (0.98–1.04)
Sex	365		
Female		1.00 (ref.)	1.00 (ref.)
Male		0.71 (0.36–1.38)	1.10 (0.67–1.80)
Ethnicity	361		
Other ethnic background		1.00 (ref.)	1.00 (ref.)
White		0.26 (0.05–1.39)	0.53 (0.11–2.48)
Employment status	356		
Fulltime		1.00 (ref.)	1.00 (ref.)
Part-time		1.33 (0.62–2.87)	0.79 (0.42–1.46)
Not working		0.87 (0.27–2.80)	1.21 (0.54–2.73)
Annual household income	356		
More than £40,000		1.00 (ref.)	1.00 (ref.)
Between £20,000 and £40,000		2.90 (1.37–6.10)	2.29 (1.25–4.16)
Less than £20,000		1.10 (0.39–3.07)	1.33 (0.64–2.74)
Age when finished education (years)	358	0.93 (0.87–0.998)	0.89 (0.84– 0.94)
Marital status	334		
Other		1.00 (ref.)	1.00 (ref.)
Married or living as married		1.16 (0.52–2.63)	1.60 (0.84– 3.05)
Communicated risk to participant (0–100)	365	1.05 (1.01–1.08)	1.03 (0.996–1.05)
BMI (kg/m^2^)	379	1.04 (0.96–1.14)	1.13 (1.05– 1.20)
Family history of diabetes	365		
Yes		1.00 (ref.)	1.00 (ref.)
No		1.25 (0.51– 3.04)	0.57 (0.31–1.05)
Smoking status	356		
Current smoker		1.00 (ref.)	1.00 (ref.)
Never smoked/ex-smoker		1.67 (0.49–5.68)	0.95 (0.42–2.14)
Baseline fruit and vegetable intake (g/day)	364	1.00 (0.999–1.001)	1.00 (0.999–1.001)
Baseline Diet Intentions (1–5)	364	1.01 (0.61–1.66)	0.81 (0.55–1.17)
Baseline response efficacy for diet	364		
Higher than 4		1.00 (ref.)	1.00 (ref.)
Less or equal 4		1.11 (0.55–2.22)	1.55 (0.90–2.65)
Baseline self-efficacy for diet	364		
Higher than 4		1.00 (ref.)	1.00 (ref.)
Less or equal 4		1.08 (0.53–2.19)	1.36 (0.79–2.35)
Baseline physical activity (KJ/kg/day)	364	0.998 (0.98–1.01)	0.995 (0.98–1.01)
Baseline Physical Activity Intentions (1–5)	364	1.05 (0.65–1.68)	0.76 (0.53–1.08)
Baseline response efficacy for physical activity	363		
Higher than 4		1.00 (ref.)	1.00 (ref.)
Less or equal 4		1.67 (0.76–3.61)	1.43 (0.82–2.50)
Baseline self-efficacy for physical activity	364		
Higher than 4		1.00 (ref.)	1.00 (ref.)
Less or equal 4		0.67 (0.34–1.34)	1.36 (0.78–2.37)

The estimates are OR and its 95% confidence interval derived from univariate multinomial logistic regressions.

Given the number of comparisons we considered the results as convincing if the 2 sided *p*-value was <0.001.None of the association were statistically significant at <0.001.

## 4 Discussion and conclusion

### 4.1 Discussion

Our study fills important gaps in the literature in the field of risk accuracy of T2D. At a time when many health systems are introducing diabetes risk assessments and knowledge about the risk of developing disease is a key element of public health campaigns, it is somewhat surprising that there is limited information on how people understand their risk of T2D after it has been communicated to them. More importantly, public health prevention programmes and campaigns are based on an assumption that one approach fits all, starting from how the risk scores are calculated through to how they are communicated and understood by the individuals being assessed. While there are several studies focusing on risk perception [32], [33], [34], studies focusing on risk accuracy are rare [15] and studies exploring factors associated with underestimation/overestimation of T2D immediately after risk information has been communicated to individuals are even rarer [15]. Based on the data collected at three different time points (before, immediately and eight weeks after provision of risk information) among middle-aged individuals, we identified that the majority of the people overestimate their absolute lifetime risk of T2D at all three time points. Immediately after provision of risk information, participants improved their risk accuracy but this was not sustained at eight weeks-post intervention, especially among those who overestimated their risk at baseline. We did not identify sociodemographic, clinical, psychological or behavioural characteristics associated with underestimation or overestimation immediately after risk was communicated to participants.

According to some theories of behaviour change, individuals need to consider themselves at (high) risk of developing the disease to adopt the recommended actions (e.g. a healthy diet, regular exercise, adherence to medication) that can prevent development of the disease [35], [36]. The importance of risk accuracy for adoption of healthy behaviours [5], [6], [7], [8] is widely accepted by professional bodies [3], [4] and provision of information on individuals’ risk of developing T2D is a central part of the recently introduced Diabetes Prevention Programme in the UK [2]. Based on our data it is possible that provision of information about lifetime risk of T2D may falsely reassure the majority in the short term and have limited impact on risk perceptions in the medium term.

While some interventions aimed at changing risk accuracy seem to be successful [15], [37], [38] others are not [39], [40], [41], [42], [43]. In this study, only 24.7% (or 41.1% when accurate was defined as ±5% of communicated risk) reported their risk accurately after receiving their risk estimate, indicating that risk perception is not as simple as recalling a number. Qualitative studies in cancer research shed some light onto why people sustain their own risk perception despite being provided with information. Possible explanations include personal or lay theories of disease and risk [44], [45], differences between laypersons’ understanding of risk information and clinical risk information [45], [46], and past experiences, expectations and beliefs [47], [48]. In our study most people overestimated their risk before receiving risk information and continued to overestimate their risk immediately after they received their risk estimate and also eight weeks later. Importantly, those who underestimated their risk at baseline improved their accuracy of risk perception immediately after intervention and sustained it eight weeks later. The PMT [18] postulates that threat appraisal (perceived vulnerability and severity) might either motivate people to adapt i.e. health-related behaviours (health protective actions) or can lead to maladaptive coping i.e. denial, fatalism, hopelessness. Having either a higher or lower inaccurate risk perception can therefore result in less adoption of health protection actions.

Our study, therefore, together with other studies [15], [49], [50], indicates that those who overestimate or underestimate their risk before receiving risk information might require different approaches for altering their risk perception. Further research is therefore needed to confirm that factors identified in aforementioned qualitative studies should be taken into account when communicating risk to participants with different baseline risk perception.

Patient-centred care and shared decision making recommendations highlight the need for clinicians to discuss the individual risk of developing disease with their patients so patients can make their own decisions regarding the prevention of the disease. Implicit to this is the belief that patients are able to understand and remember their risk accurately. Interestingly, theories of behaviour change [5], [6], [7], [8] incorporate risk accuracy as an important component of behaviour change but do not incorporate factors that influence the accuracy of risk perception. If risk information is to continue to be routinely communicated to patients by clinicians, it is important to understand whether patient characteristics determine whether once the risk information is communicated, it is also perceived accurately. In this study we did not identify sociodemographic, clinical, psychological or behavioural characteristics associated with underestimation or overestimation immediately after risk was communicated to participants and a similar pattern was also observed when underestimation and overestimation was measured eight weeks after provision of risk information (results not shown). The results also remained the same when we adjusted all analyses for the modelled risk score (results not shown).

This is different from previous studies in which certain demographic characteristics such as age [15], sex [15], ethnicity [51] education [51], and clinical characteristics including BMI [15], [51], as well as psychological and behavioural characteristics such as motivation to engage in healthy behaviours [52] and smoking status [51] were associated with risk accuracy. In the present study, there was an indication that level of education and BMI might be associated with inaccuracy; however this did not reach our threshold for statistical significance. Given the sample size in the present study, it is possible that this study was not sufficiently powered to identify small but clinically meaningful associations found in studies with larger sample size [15], [51]. Previous studies have identified that dispositional optimism/pessimism [53], numeracy [54], [55] and health literacy [56] were associated with risk accuracy, but neither were measured in this study. Future research is needed with adequately powered studies to replicate previous findings and also explore other possible determinants of misperception of T2D risk [44], [47].

Results of the present study need to be interpreted in light of its other limitations. Participants were from one location in the United Kingdom, mostly white middle-aged adults. Future research should explore whether the results of the present study will be replicated in different ethnic and age groups. Additionally, the study sample was relatively small, and may not have been sufficiently powered to identify small but clinically meaningful differences. Lastly, we assessed perceived risk using a single-item measure, operationalizing perceived risk in epidemiological terms on a scale 0–100. At present, it is recognized that understanding risk is a dynamic and challenging process [57] and using a simple numerical measure of risk perception does not always reflect the complexity of an individuaĺs understanding of their risk [45], [48]. In addition, we did not provide participants with the option of ‘Do not know’ [58].

### 4.2 Conclusion

Our study indicates that understanding a received risk estimate is challenging for most participants with many continuing to have inaccurate risk estimates after receiving the estimate. Those who overestimate or underestimate their T2D risk before receiving risk information might require different approaches for altering their risk perception. Those delivering diabetes prevention programmes should consider how information about risk is provided in order to optimise efforts to reduce disease incidence. If the provision of T2D risk information remains a central part of individual-based T2D preventive strategies, then further research is needed to identify the best way to present T2D risk. Future research is also needed with sufficiently powered studies to replicate our findings and explore other potential determinants of risk accuracy such as numeracy, knowledge, dispositional optimism, past experiences, expectations and beliefs.

### 4.3 Practice implications

Presently, there is a strong enthusiasm for the use of risk estimates in daily clinical practice and as part of population prevention strategies [2], [3], [4], even though their utility at the individual level has been questioned by some scholars [59] and empirical evidence [11], [12]. In the present study, we only focused on a small fraction of more complex risk communication process, namely risk accuracy. This study defined risk perception of T2D and accuracy in epidemiological terms that might be different than individuaĺs understanding of risk [44], [45]. Therefore, when clinicians communicate risk of developing disease to their patients, they should take into account individual’s personal and family experiences and beliefs about i.e. T2D rather than simply asking them whether they understood the communicated number to them.

## Conflicts of interest

None.

## Submission declaration and verification

We declare that the work described here has not been published previously, it is not under consideration for publication elsewhere, its publication is approved by all authors and tacitly or explicitly by the responsible authorities where the work was carried out, and that, if accepted, it will not be published elsewhere in the same form, in English or in any other language, including electronically without the written consent of the copyright-holder.

## Contributors

The contributions of authors to the manuscript are as follows: a) study concept and design: Silarova, Douglas, Usher-Smith, Griffin; b) acquisition, analysis or interpretation of data: Silarova, Douglas, Usher-Smith, Godino, Griffin; c) drafting of the manuscript: Silarova, Douglas; d) critical revision of the manuscript for important intellectual content: Silarova, Douglas, Usher-Smith, Godino, Griffin; e) obtained funding: Griffin.

All authors have approved the final article.

## Role of the funding source

The DRCT trial was supported by The Medical Research Council (MC_U106179474), and conducted at the MRC Epidemiology Unit in Cambridge, UK. Protocol development was supported by the European Union (Integrated Project LSHM-CT-2006-037197 in the Framework Programme 6 of the European-Community) and the National Institute for Health Research (RP-PG- 0606-1259). BS was supported by the Medical Research Council [MC_UU_12015/4]. JUS is supported by a National Institute for Health Research Clinical Lectureship. SG is a National Institute of Health Research Senior Investigator and member of the NIHR School for Primary Care Research. The study funder(s) had no role in the study design; in the collection, analysis, and interpretation of data; in the writing of the report; and in the decision to submit the article for publication. All researchers state that they are independent from funders.
